# Successful treatment of rapid growing mycobacterial infections with source control alone: Case series

**DOI:** 10.1016/j.idcr.2021.e01332

**Published:** 2021-11-06

**Authors:** Igor Dumic, Larry Lutwick

**Affiliations:** a Mayo Clinic College of Medicine and Science, Rochester, MN, USA; b Division of Hospital Medicine, Mayo Clinic Health System, Eau Claire, WI, USA; c Division of Infectious Disease, Mayo Clinic Health System, Eau Claire, WI, USA

**Keywords:** Non tuberculous mycobacteria, Source control, Skin and soft tissue infection, Bloodstream infection, *Mycobacterium abscessus*, *Mycobacterium chelonae*, *Mycobacterium fortuitum*

## Abstract

Rapid growing mycobacteria have been increasingly recognized as pathogens, both in immunocompromised and immunocompetent population, and their incidence has increased over the last decade significantly. Pulmonary infections are the most common, however, any organ can be affected. The treatment of these infections is costly, prolonged, and often antimicrobial resistance poses a significant challenge to a successful outcome. The source control together with antimicrobials is the cornerstone of treatment. We report a case series of 3 patients with extrapulmonary rapid growing mycobacterial infections in whom the successful treatment was achieved with source control alone.

## Introduction

Rapidly growing mycobacteria (RGM) are non-tuberculous mycobacteria (NTM) that can form mature colonies on agar plates within 7 days [1]. They have been increasingly recognized as pathogens in both immunocompetent and immunocompromised populations, and their incidence has surpassed that of *Mycobacterium tuberculosis* in developed countries. The most common RGM that cause human disease are *Mycobacterium abscessus*, *Mycobacterium chelonae* and *Mycobacterium fortuitum* (complex) [2]. They are ubiquitous and are readily found in water, dust and soil and have been implicated as nosocomial pathogens capable of causing outbreaks in healthcare facilities [3]. Virtually any organ can be affected, however, pulmonary infections are the most common. Extrapulmonary infections including skin and soft tissue infections and bloodstream infections are less frequent [4], [5]. Treatment of these infections is usually prolonged and includes combination of antimicrobials and source control if possible. Particularly challenging for treatment are *M. abscessus* infections because of multidrug resistance, need for prolonged therapy and development of side effects [6]. Successful treatment of extrapulmonary RGM infections with sole source control without antimicrobials has not been commonly reported.

## Case presentation

### Case 1

A 72 year old woman with medical history of well controlled hypertension and hypothyroidism underwent minimally invasive aortic valve replacement surgery for aortic stenosis. Postoperatively, she did well and was discharged with retained epicardial pacemaker leads, having received perioperative cefazolin. She did well for approximately two months but then developed redness, swelling and purulent discharge at the incision site in right inframammary area at the working port site. She had no fever, chills or systemic signs of infection, however, given clinical findings, she was taken to operating room for debridement. Purulent discharge was found superficially subcutaneously, and atrial and ventricular epicardial pacemaker wires were removed without complication. Blood and wound cultures were sent. One wire was covered by granulation tissue inside the chest wall and was unable to be removed, remaining *in situ*. The wound cultures were initially negative but did yield an acid fast bacillus, and she had been empirically started on amoxicillin-clavulanic acid and trimethoprim-sulfamethoxazole, completing only 10 of 21 days of therapy due to gastrointestinal side effects.

In post hospital discharge follow up visit two weeks later, she was noticed to have again purulent drainage and was seen by Infectious Diseases and re-admitted again for debridement and wound exploration. *Mycobacterium abscessus* group had been identified as the acid fast bacilli (AFB) in the initial culture. Again, the patient did not have systemic signs of infection or leukocytosis. Her wound was approximately 8.0 centimeters deep and contained purulent material again.

Surgically, extensive incision and excisional debridement of the working port site tract, which was found to extend into the pleural space, and the remaining temporary pacing wire was removed. The final wound was 7.5 cm long and 3 cm wide and 8.5 cm deep extending into the pleural space. The entire tract was removed. Surgical pathology showed acute inflammation and granulation tissue without granulomas, and AFB stains were negative.

Blood cultures were drawn, and following debridement, wound cultures were sent. Following that, her inflammatory markers improved with C-reactive protein decreasing from 15.8 mg/L to 1 mg/L (normal value< 8 mg/L), and erythrocyte sedimentation rate decreased from 58 to 12 mm/h (normal value below 20). The wound cultures again grew acid fast bacilli, characterized as *Mycobacterium abscessus* group by Matrix-assisted laser desorption/ionization time-of-flight (MALDI-TOF, Mayo Clinic Laboratory, Rochester, MN). The antimicrobial resistance profile showed sensitivity to only amikacin and resistance to imipenem, doxycycline, cefoxitin, linezolid, and trimethoprim sulfamethoxazole. Test for inducible clarithromycin resistance was positive and the organism was found to be sensitive to clofazimine (done at Microbiology Laboratory at National Jewish Hospital, Denver, CO, USA).

Due to the medication cost, potential side effects and an upcoming family wedding, the patient opted against antimicrobial therapy. Over 6 weeks, her wound completely healed. Follow up CT imaging 2 months later revealed no evidence of persistent infection. 30 months later, she continued to do well without evidence of recurrence in the absence of specific antimicrobial therapy with normal inflammatory markers.

### Case 2

A 41 year old woman with history of untreated bipolar disorder, post-traumatic stress disorder (PTSD), and substance abuse (intravenously methamphetamine) was admitted for 4 days of generalized weakness, cough productive of yellowish sputum and shortness of breath. Her symptoms started four days prior to admission. CT of the chest was significant for bilateral patchy and scattered areas of airspace opacities with multiple cavitary nodules. Blood cultures were drawn, and she was empirically started on antimicrobials with levofloxacin for community acquired pneumonia and sepsis. Blood cultures were positive for methicillin sensitive *Staphylococcus aureus* (MSSA) after 12 h of incubation, and 8 blood cultures were positive over 4 days before clearance. Transthoracic echocardiogram showed tricuspid anterior leaflet valve vegetation measuring 1 × 0.6 cm. Three days after admission, because of persistent lower back pain, imaging revealed vertebral osteomyelitis and a psoas abscess which was surgically drained and also grew MSSA.

She was transitioned to oxacillin with improvement in laboratory and radiological findings and was discharged with peripherally inserted central catheter (PICC) to skilled nursing facility to complete total of 8 weeks. However, despite therapy, the vegetation on the tricuspid valve progressed and posterior leaflet was affected in addition to previously known anterior leaflet infection. She was continued on anti-staphylococcal therapy with oxacillin and underwent tricuspid valve repair without complications. Intraoperative valvular cultures were negative. However, three days postoperatively she developed fever, and blood cultures peripherally and from the PICC line were positive for *M. chelonae* (MALDI TOF, Mayo Clinic Laboratories, Rochester, MN, USA) after 4 days; followup cultures were positive from the PICC, while peripheral blood cultures remained negative. The PICC line was removed, and the tip grew the same organism, greater than 15 colony forming units on semiquantitiative culture. Eventually, the organism was reported sensitive to amikacin, tobramycin and clarithromycin, intermediate to imipenem and linezolid and resistant to cefoxitin, moxifloxacin, doxycycline, minocycline and moxifloxacin.

Subsequently, the patient did not have any recurrence of fever, chills and remained with normal white blood cell count. Given recent tricuspid valve replacement, trans-esophageal echocardiogram was obtained that showed no vegetation. Because of rapid clearance, no specific therapy was given. Serial blood cultures were repeated and remained negative with several sets obtained over three months. She continuing to do well without antimicrobials.

### Case 3

A 62 year old woman was admitted for redness, swelling, purulent discharge, and pain in her right upper chest wall, at the site of a Port-A-Cath. She was a lifelong Wisconsinite, worked as a cook, and had not traveled outside the state over the last year. She had no pets, smoked half a pack per day, and did not drink any alcohol or use illicit drugs. Her past medical history was significant for Crohn's disease requiring multiple bowel resections including subtotal colectomy with ileostomy placement, recurrent episodes of hypomagnesemia, dehydration and acute kidney injury due to high output ileostomy, and stage III chronic kidney disease due to focal segmental glomerulosclerosis. Crohn’s disease was in remission on vedolizumab.

One year earlier, she had a Port-A-Cath inserted for intravenous infusions and recurrent, chronic hypomagnesemia for which she received intravenous magnesium replacement four times a week. Three days before admission, she was found to have right upper chest wall cellulitis, peripheral blood cultures were sent, and treatment initiated with ceftriaxone 2 g daily via Port-A-Cath. Despite this, the redness, swelling and pain worsened, and she developed purulent discharge at the site of the requiring admission. Her antimicrobials were broadened to cefepime and vancomycin, vedolizumab was held, and Port-A-Cath was removed. Catheter tip cultures and another set of peripheral blood culture were sent. On day two of admission (5 days after initial peripheral blood cultures were sent) blood cultures grew an AFB. The next day, a second set of peripheral blood cultures and catheter tip cultures grew AFB as well. Vancomycin and cefepime were discontinued, and stigmata of cellulitis receded. She remained hemodynamically stable throughout her hospital stay and remained afebrile. Subsequent blood cultures (after removal of the catheter) remained negative. Acid fast bacillus was determined to be *Mycobacterium chelonae* (Mayo Clinic Laboratories, Rochester, MN, USA) sensitive only to clarithromycin, linezolid, tobramycin and amikacin. Medication cost was prohibitive to the patient, and she decided to proceed with observation off of antimicrobials. One year after this episode her blood cultures remain negative, and the patient did not develop any symptoms or signs of recurrent infection.

## Discussion

Of all NTM infections in the United States, only *Mycobacterium avium complex* (MAC) is more common than *M. abscessus* and *M. chelonae* [7]. *M. abscessus* was first described in 1952 as a cause of knee abscess [8]. Since then taxonomy has evolved, and what was considered the same species was reclassified in 1992, when *M. abscessus* and *M. chelonae* became separate species. In 2013, *M. abscessus* complex was further subspeciated to include subspecies *abscessus*, *massilliense* and *bolletti* [7], [9]. Among these,*M. abscessus* subsp. *bolletti* is the least isolated human pathogen. The main difference between the other two subspecies (*M. abscessus* subsp. *abscessus* and *M. abscessus* subsp. *massillience*) is the erm (41) gene pattern that confines inducible resistance to clarithromycin [7]. The presence of functional erm (41) gene (ribosomal methyltransferase) in *M. abscessus* subsp. *abscessus* results in inducible resistance to macrolides (susceptible on day 3 but resistant of day 14 of incubation). On the other hand, M. *abscessus* subsp. *massillience* has nonfunctional erm (41), so inducible resistance to clarithromycin does not occur which has significant implication for therapy and consequently the outcome of these infections [7], [10].

Skin and soft tissue infections (SSTI) caused by *M. abscessus* range from mild superficial and localized infection to deep tissue infections. The two major mechanisms implicated in acquisition of these infections are either direct contact with contaminated material through traumatic wound, surgical incision and/or other environmental exposures, or the skin might be infected during disseminated forms of disease [7], [9]. We believe that in patient 1, infection was nosocomial, and probably temporary pacemaker leads were contaminated. This is supported by the fact that infection persisted and recurred despite debridement until pacemaker lead that was retained was removed. In addition to surgical site infection, SSTI due to RGM were reported in relation to patients who underwent liposuction, mesotherapy, intramuscular injections and acupuncture [10].

The guidelines of Infectious Disease Society of America (IDSA) and American Thoracic Society (ATS) recommend therapy based on the results of medication susceptibility testing. It should include macrolide based regimen (if susceptible) with addition of another one or two IV medications such as imipenem, amikacin or cefotaxime [11]. Duration of therapy is based on expert opinions and retrospective studies, while there are no clinical trials that specifically address this question. Duration of therapy is different for extra pulmonary infections that are considered easier to treat than pulmonary infection; pulmonary infections are frequently chronic and incurable despite prolonged therapy with intravenous agents [11]. For skin and soft tissue infection, total of 4–6 months of therapy is recommended following at least 2 weeks of initial parenteral therapy [11], [12]. Given high cure rate of extra pulmonary infections, in comparison to pulmonary infection, it was postulated that majority of extra pulmonary infections are caused by macrolide sensitive *M. massillience* or C28 sequevar of *M. abscessus,* which is sensitive to clarithromycin. Retrospective study of 22 patients from South Korea with extra pulmonary NTM infections demonstrated that all 3 patients who had unfavorable response to therapy were infected with *M. abscessus* isolate with an intact erm (41) gene, therefore conferring resistance to macrolide [12].

Prolonged therapy is associated with significant side effects from antimicrobials. Emerging Infections network (EIN) study group collected 65 cases from 16 US states in 6 months period. Among these 34 patients (62%) developed side effects. The most common side effects were nausea and vomiting. The most common medications to cause side effects was amikacin in 30% of cases and tigecycline in 18% [5]. Among patients who developed side effects while receiving amikacin and tigecycline, 51% and 36% had to adjust or stop therapy, respectively. In addition to significant side effects as described, a considerable barrier to therapy is the cost of medications as well. It is estimated that cost of therapy for NTM ranges from $398–70,971 (average $19,876) with infection with *M. abscessus* being much more costly (average $ 47,240) [13], [14]. The cost precluded our patient from Case 1 to receive therapy.

In addition to antimicrobial therapy, surgical resection and source control is recommended in case of extra pulmonary *M. abscessus* and *M. chelonae* infection if there is abscess formation, extensive involvement or when drug therapy due to resistance or cost issue is difficult [14]. Source control with removal of foreign material is mandatory. To the best of our knowledge this is the first case report of successful treatment of SSTI due to multidrug resistant *M.abscessus* with only source control.

One case report demonstrated successful treatment of *M. chelonae* peritoneal dialysis catheter related infection with use of thermal therapy in addition to antimicrobials [15]. Thermal sensitivity of *M. chelonae* and inability to grow on higher temperature was the basis of the success of this mode of therapy.

Up to 60% of all in hospital bacteremia are due to vascular access devices, and central line associated blood stream infections are commonly encountered complications [16]. Risk factors associated with development of blood stream infections (BSI) due to RGM have been described and include: neutropenia, human immunodeficiency infection, chemotherapy, therapy with corticosteroids, hemodialysis, surgery and total parenteral nutrition [1], [17], [18]. One study from South Carolina [17] demonstrated malignancy to be the most common risk factors (45.5% cases) followed by chronic gastrointestinal pathology (inflammatory bowel disease and chronic pancreatitis) in 27.3% cases. The same study showed that 78.8% of patients have received antibacterial therapy within 30 days of onset of RGM BSI. Apart from recent surgery and prolonged anti-staphylococcal therapy, our second patient had no other traditionally recognized risk factors and was immunocompetent.

Due to their ability to live in water pipes, tolerate extreme environments and the fact that they are resistant to most commonly used disinfectants, RGM are “ideal candidates” for nosocomial infections. RGM additionally can form biofilms, which is a crucial factor in pathogenesis of RGM BSI. In fact, Hall-Stoodley and co-authors demonstrated that *M. chelonae* can form biofilm as soon as 48 h from contact with catheter surface [19]. Embedded in dense biofilm, rapid growers become resistant, so antimicrobials alone are unable to eradicate infection, and removal of catheter is recommended [1].

There are no randomized control trials done to provide recommendation about the most appropriate antimicrobial therapy choice and duration. However, a few studies demonstrated that relapse rates of BSI are higher and cure rates lower if the catheter is retained [16], [20]. Duration of at least 4 weeks of therapy with removal of catheter provides cure in more than 90% of cases [1], [17], [20]. BSI due to RGM overall have lower mortality than BSI due to gram positive bacteria or fungal pathogens [1].

## Conclusion

In these three cases, we demonstrated the importance of adequate source control for successful treatment of rapidly growing SSTI and BSI. It seems that some immunocompetent patients can clear infection on their own in the absence of antimicrobial therapy, and this approach should be considered in patients in whom the cost of therapy is prohibitive, risk of side effects considerable and/or lack of options complicates treatment due to drug resistance.

## Ethical approval

Not required.

## Consent

Obtained.

## Funding

10.13039/100000871Mayo Clinic.

## Conflict of interest

None.
